# Anti-inflammatory and cytotoxic effect of lycopene and raspberry in-situ gel

**DOI:** 10.6026/97320630018645

**Published:** 2022-07-31

**Authors:** V Mukundh Chaithanya, TN Uma Maheswari, S Rajesh Kumar

**Affiliations:** 1 Saveetha Dental College and Hospitals, Saveetha Institute of Medical and Technical Sciences (SIMATS), Saveetha University, Chennai, India

**Keywords:** Lycopene, Raspberry, antioxidant, anti-inflammatory

## Abstract

Preparation of an in-situ gel containing lycopene and raspberry plant formulation and analysis of its anti-inflammatory and antioxidant effects is described. Lycopene is known for its anticancer and antioxidant activity. It induces apoptosis, thereby reducing the cancer cells, and also reduces the injury of cells due to oxidative activity. Similarly, raspberry also contains antioxidants properties which can help to reduce oxidative stress and chronic inflammation. This study includes extracts containing 25% of Raspberry and 10% lycopene, carboxymethyl cellulose, hydroxypropyl methyl cellulose, Carbopol, sodium chloride, and distilled water. This in-situ gel was then tested for antioxidant and anti-inflammatory assay using DPPH (2, diphenyl 1-picryl- hydrazyl- hydrate) and bovine serum albumin (BSA), antioxidant assay revealed that the inhibition percentage was more with 50 µL (61.3) of gel and anti-inflammatory assay showed significant results with 10 µL (90.2) of gel. In-situ gel containing lycopene and Raspberry has significant anti-inflammatory and antioxidant activity.

## Background:

In-situ gel production is one of the most complex processes as various biopolymers are used. Lycopene and Raspberry are known for their antioxidant and anti-cancerous properties. Lycopene, due to its highly lipophilic nature with its protecting mechanism of tissues from the lipid -peroxidation activity, can help to prevent injury, and it is also the most antioxidant among 500 carotenoids present, especially in comparison with beta-carotene, also studies with evidence of pro-oxidative side effects is also seen less in lycopene [2-32]. The lycopene, when consumed directly from tomato, is known as active or bio-lycopene, which has certain anti-inflammatory properties helping to reduce chances of chronic and systemic diseases, the anticancer property of lycopene has proven in various studies to help to reduce even the moderate to advanced stages of cancers and help in the prevention of liver, prostate and other cancers of the body [4-20]. In-situ gel preparation was done for the purpose of treating disease and its better drug delivery application. They have known for their gelling attribution and long-standing property, which is known as residence timing; this enables the drug to have more duration of action in the specified location along with better absorption [23, 29]. The flowing capacity or the pourability of the gel is due to polymers such as Hydroxypropyl methylcellulose (HPMC) and Sodium alginate, which is present within the formulation [9, 11]. The advantage of the in-situ bioadhesive gel is the availability at the site and residence time, which in turn is better than a conventional gel medium [18, 21]. Previously our team has a rich experience in working on various research projects across multiple disciplines [6, 7, 8, 10, 14, 16, 19, 26, 27, 28, 30, 34, 35, 36, 37, 38]. Therefore, it is of interest to document the anti-inflammatory and cytotoxic effect of lycopene and raspberry in-situ gel.

## Materials and methods:

### Materials used:

The materials used in this study include extracted compounds containing 10% of lycopene and 25% of raspberry concentration; these materials were acquired from authentic biomaterial sellers and are used in this process of making an in-situ intraoral gel. Other chemical polymers used for making this in-situ gel include Carboxymethyl Cellulose (CMC) (gelation compound-water retention property), Hydroxypropyl methyl Cellulose (HPMC) (film coating agent), Carbopol (stabilizing compound) and Sodium chloride (NaCl) (as a preservative) all which were obtained from a chemist (Figure 1 - see PDF).

### Preparation of lycopene and Raspberry in-situ gel:

A beaker of 15 ml of distilled water is taken, and 1.5gm of lycopene extract is mixed with 1.5 gm of raspberry extract; these extracts were then heated thoroughly until the solution is reduced to about 5mL of concentration in order to reduce the water and increase the concentration of extraction. Then the solution is cooled down, this is followed by the addition of 2.50 grams of Carbopol, 0.50 gm of HPMC, and 0.95gm of NaCl, and 0.50gm of CMC, all the contents are stirred thoroughly in between the addition of the other (Figure 2 - see PDF).

### Determination of antioxidant activity:

The in-situ gel of lycopene and Raspberry is then subjected to DPPH assay, the lycopene, and raspberry extracts are then subjected to 5 different concentrations of 10 µL, 20 µL, 30 µL, 40 µL, 50 µL in a solution containing 2 mL of DPPH, the solution is then kept for incubation for about 15-20 mins (dark incubation), these five samples are further subjected spectrophotometry and analyzed for inhibition levels.

### Determination of anti-inflammatory activity:

The in-situ gel of lycopene and Raspberry is subject to Bovine serum albumin assay (BSA), the solution containing lycopene and raspberry gel is subjected to 5 different concentrations of 10 µL, 20 µL, 30 µL, 40 µL, 50 µL in a solution containing 1mL of BSA, the solution is maintained at a room temperature for 10 mins, this is followed by boiling of the contents at 55 degree Celsius for 10-15 mins, this solution is then subjected to spectrophotometry for inhibition level analysis. (Figure 4 - see PDF)

## Results and Discussion:

The results of this study have shown that the in-situ gel preparation of lycopene-raspberry gel has a better Inhibition action in both antioxidant and anti-inflammatory assay analysis. Spectrophotometry readings of antioxidant assay revealed that 50 µLl of lycopene-raspberry extracts have more absorption percentage of about 41% (Figure 5 - see PDF). In comparison, anti-inflammatory assay has revealed that Lycopene-raspberry in-situ gel concentration of about 10 µL has given an absorption percentage of about 79.7% (Figure 6 - see PDF). Based on the spectrophotometry analysis results, the inhibition percentage of lycopene and Raspberry has been calculated to test the real-time activity of the in-situ gels, the antioxidant activity has shown the highest inhibition percentage in relation to 61.3% in 50µL concentration of in-situ gel of lycopene-raspberry (Figure 7 - see PDF), and the anti-inflammatory assay has revealed that 90.2% of inhibition percentage has seen in 10µL concentration of lycopene-raspberry in-situ gel when compared to other levels (Figure 8 - see PDF).

Studies done by Mallery et al. have shown that raspberry bioadhesive (in-situ) has shown a reduction in pre-malignant lesions and antioxidant activity, indicating its better inhibition activity which is also in line with our study; one of the most significant of in-situ formulation in the study is the complete absence of any adverse or deleterious effects in the usage of in-situ gels vs normal gel [13,17].While other studies done by Bansal et al. has shown that lycopene along with a combination of polymers (in-situ gel formers such as tween 40, HPMC, etc.) has shown better results due to the increase in patients’ compliance, better adhesive property, reduced effects of UV due to gel application which is also one of the disadvantages of conventional gels [2,16]. Certain studies done by Bignotto et al. has shown that the lycopene has a high significant antioxidant property in the dose of 25mg/kg, reducing the risk of cancer and also the anti-inflammatory action in which the injury to the liver cells was reducing the pain [1,3]. While studies did by padmavathy et al. has revealed that in-situ gel formulation has good sustainability (sustained-release), more patient's compliance (less irritation), ease of administration and good retention properties [12, 33]. Our institution is passionate about high-quality, evidence-based research and has excelled in various fields [5, 15, 22, 24, 25, 31, 39]. We hope this study adds to this rich legacy.

## Conclusions:

The present study was done to formulate intraoral in-situ gel using modern technique and spectrometry analysis showing significant antioxidant and anti-inflammatory properties. Hence this study concludes that a combination of lycopene and raspberry formulation has better antioxidant and anti-inflammatory properties.
